# Efficacy and safety of iGlarLixi versus IDegAsp in people with type 2 diabetes inadequately controlled with basal insulin: A systematic literature review and network meta‐analysis of non‐Asian studies

**DOI:** 10.1111/dom.16360

**Published:** 2025-04-02

**Authors:** Philip Home, Felipe Lauand, Khier Djaballah, Xuan‐Tony Li, Khadija Hafidh, Roopa Mehta, Khadra Faraoun, İnan Anaforoğlu, Paul Serafini, Mir‐Masoud Pourrahmat

**Affiliations:** ^1^ Translational and Clinical Research Institute Newcastle University Newcastle upon Tyne UK; ^2^ Sanofi Paris France; ^3^ Sanofi Bridgewater New Jersey USA; ^4^ Department of Internal Medicine, Diabetology Unit, Rashid Hospital Dubai Health Authority Dubai United Arab Emirates; ^5^ Departamento de Endocrinología y Metabolismo, UIEM Instituto Nacional de Ciencias Médicas y Nutrición, Salvador Zubirán México City Mexico; ^6^ Faculty of Medicine of Oran Université Oran 1 Oran Algeria; ^7^ Faculty of Medicine Mehmet Ali Aydınlar Acıbadem University Istanbul Turkey; ^8^ Evidinno Outcomes Research Inc Vancouver British Columbia Canada

**Keywords:** fixed‐ratio combination, iGlarLixi, insulin therapy, network meta‐analysis, type 2 diabetes

## Abstract

**Aims:**

To estimate the relative treatment effect of iGlarLixi (a fixed‐ratio combination of insulin glargine 100 U/mL plus lixisenatide) versus premixed insulin IDegAsp (insulin degludec plus insulin aspart) in people with type 2 diabetes (T2D) who advanced from basal insulin to iGlarLixi or IDegAsp in non‐Asian studies.

**Materials and Methods:**

Randomized controlled trials (RCTs) were identified in a systematic review by searching Embase (including congress abstracts from 2021 to 2023), MEDLINE® and CENTRAL on 10 October 2023. Treatment outcomes from non‐Asian RCTs for people with T2D previously treated with basal insulin, who switched to iGlarLixi or IDegAsp, were compared using a network meta‐analysis (NMA). Data analysis was performed using R, version 4.0.2.

**Results:**

The NMA included four RCTs (*N* = 2535). The results of the NMA showed that iGlarLixi (*n* = 810) was associated with a significantly greater reduction in HbA1c versus IDegAsp (*n* = 454) (mean difference [MD]: −0.39 [95% credible interval, CrI: −0.58, −0.21] %‐units). iGlarLixi was also associated with a significantly greater likelihood of achieving an HbA1c of <7.0% (risk ratio: 1.42, 95% CrI: 1.18, 1.71). A greater reduction in postprandial glucose was observed with iGlarLixi versus IDegAsp (MD: −1.38 [95% CrI: −2.15, −0.63] mmol/L). A body weight benefit that favoured iGlarLixi versus IDegAsp was documented (MD: −1.54 [95% CrI: −2.26, −0.84] kg). Hypoglycaemia evaluation was inconclusive due to definitional differences between trials.

**Conclusions:**

Once‐daily iGlarLixi was associated with superior blood glucose control and body weight benefit compared with IDegAsp in insulin‐experienced populations with T2D in non‐Asian RCTs.

## 1 INTRODUCTION

In 2021, it was estimated that 537 million adults (aged 20–79 years) had diabetes, with over 90% of these having type 2 diabetes (T2D).^1^ Type 2 diabetes is a chronic, progressive disease characterized by the continuing loss of islet β‐cell function.^2^ The American Diabetes Association (ADA) and the European Association for the Study of Diabetes (EASD) consensus statements recommend a glycated haemoglobin (HbA1c) target of <7.0% for most adults with T2D.^3^ With progression, the introduction of injectable therapies is usually required for people with inadequate glycaemic control when using oral antihyperglycaemic drugs (OADs). For people with T2D that continue to have an HbA1c above target, despite the use of basal insulin, therapy advancement with the addition of a glucagon‐like peptide‐1 receptor agonist (GLP‐1 RA) or prandial insulin may be recommended, in particular to improve postprandial glucose (PPG) control.^4^, ^5^ These combination therapies can be delivered via separate injections or, for user convenience, in one combined preparation.^6^

iGlarLixi, a once‐daily, fixed‐ratio combination of insulin glargine 100 U/mL (iGlar; a once‐daily basal insulin) and the GLP‐1 RA, lixisenatide (Lixi), has been shown to result in improved glycaemic control versus its individual components in both insulin‐naïve and insulin‐experienced people with T2D.^6^, ^7^, ^8^ iGlarLixi has also demonstrated improved glycaemic control, weight benefit and reduced risk of hypoglycaemia versus the premixed insulin, biphasic insulin aspart 30/70 (BIAsp 30), in people with T2D suboptimally controlled on basal insulin plus OADs.^9^ The combination of basal insulin and GLP‐1 RA provides effective glycaemic control and mitigates body weight gain and the risk of hypoglycaemia that are associated with insulin therapy.^6^

IDegAsp is a once‐ or twice‐daily premixed insulin, combining prandial insulin aspart (IAsp) with basal insulin degludec (IDeg; a long‐acting insulin analogue [>24 h]) at a ratio of 30:70.^10^ Phase 3 clinical trials have shown that IDegAsp was non‐inferior in terms of glycaemic control in comparison with basal insulin glargine,^10^ premixed insulin BIAsp 30^11^ and a meal‐time plus basal‐bolus regimen (IAsp and IDeg in separate injections).^2^, ^12^ In addition, IDegAsp demonstrated a body weight benefit and less hypoglycaemia versus BIAsp 30,^11^ and a similar body weight change and increased rate of hypoglycaemia versus iGlar.^10^

The clinical outcomes of iGlarLixi and IDegAsp have previously been compared in people with T2D uncontrolled on prior OADs or basal insulin in both global and Japanese populations in a systematic literature review (SLR) and subsequent network meta‐analysis (NMA), based upon eight randomized‐controlled trials (RCT).^13^ The results suggested that iGlarLixi offered improvements in glycaemic efficacy and body weight change versus IDegAsp, although with increased rates of gastrointestinal intolerance, and that iGlarLixi may be beneficial in people with T2D who require both basal and meal‐time intervention. The comparison of rates of hypoglycaemia was inconclusive due to differences in the definition among the included trials.

The recent phase 3b Soli‐D RCT compared the efficacy and safety of iGlarLixi versus IDegAsp in insulin‐naïve Chinese people with T2D.^14^ The results showed that iGlarLixi was associated with both non‐inferior and superior reductions in HbA1c, a body weight benefit and lower hypoglycaemia event rates versus IDegAsp.^14^ There are currently no direct comparisons in non‐Asian people who have received treatment with basal insulin. Insulin‐naïve, Asian populations may exhibit different clinical characteristics from those of insulin‐experienced, non‐Asian populations.^15^ In addition, the ratio of iGlar and Lixi in the iGlarLixi pen‐injector used in Chinese and Japanese populations differs from the ratio used in non‐Asian populations.^16^, ^17^ This allows for the optimization of the Lixi dose whilst accommodating different insulin dose requirements in the respective populations.^9^, ^15^, ^16^, ^17^ Therefore, an updated NMA was indicated to compare iGlarLixi versus IDegAsp in non‐Asian studies comprising insulin‐experienced populations to support the use of iGlarLixi in these populations.

Accordingly, the objective of the present study was to update the previous systematic literature review and conduct an NMA to estimate the relative treatment effect of iGlarLixi versus IDegAsp in populations with T2D inadequately controlled with basal insulin.

## 2 MATERIALS AND METHODS

### 2.1 Systematic literature review

Searches were conducted in MEDLINE®, the Cochrane Controlled Register of Trials (CENTRAL) and Embase (for journal articles and congress abstracts from the ADA, EASD, American Association of Clinical Endocrinology [AACE] and International Diabetes Federation [IDF] between 2021 and 2023) on 10 October 2023 using predefined search strategies (Tables S1–S3) to identify recently published RCTs.

The eligibility criteria for the SLR included RCTs evaluating iGlarLixi or IDegAsp compared with OADs, meal‐time insulin (rapid‐acting and short‐acting insulin), basal insulin (intermediate‐acting and longer‐acting insulins), premixed insulin (e.g., BIAsp 30) or GLP‐1 RA in populations with T2D inadequately controlled with previous glucose‐lowering therapies. Only non‐Asian RCTs were included.

Treatment outcomes from non‐Asian studies in populations with T2D who advanced from basal insulin to iGlarLixi or IDegAsp were compared using an NMA.

### 2.2 Outcomes

Efficacy outcomes included change from baseline in HbA1c, the proportion of people reaching <7.0% HbA1c, venous fasting plasma glucose (FPG) tested in a clinic, pre‐ and post‐breakfast, lunch and dinner, self‐monitored plasma glucose (SMPG), bedtime SMPG, PPG, PPG excursion, insulin dose and change in body weight.

Adverse effect outcomes compared by the NMA included rates of documented hypoglycaemia (between <54 and <56 mg/dL), severe hypoglycaemic events, the risk of at least one adverse event (AE) and serious AEs (SAEs).

### 2.3 Data analysis

Bayesian NMAs were conducted according to the National Institute for Health and Care Excellence (NICE, UK) Decision Support Unit (DSU) Technical Support Document 2 (TSD 2).^18^ Network meta‐analyses were performed using three‐chain Markov Chain Monte Carlo simulations implemented in OpenBUGS (v3.2.3; Medical Research Council, Biostatistics Unit, Cambridge, UK). Separate Bucher indirect treatment comparisons were conducted, comparing iGlarLixi with once‐daily and twice‐daily IDegAsp as sensitivity analyses. Data analysis was performed using R (version 4.0.2; The University of Auckland, New Zealand). Model selection and inconsistency were evaluated using the methodology recommended by the National Institute for Health and Care Excellence (NICE; Table S4).^18^

For each NMA, point estimates comparing iGlarLixi and IDegAsp were calculated with 95% credible intervals (CrI). For continuous outcomes, the mean difference (MD) between the two treatments was calculated, whereas for dichotomous outcomes, the risk ratio was reported. The statistical significance of the difference in risk ratio or MD between each pair of arms was assessed at the 0.05 level by determining whether the 95% CrI included 0 for continuous outcomes and 1 for dichotomous outcomes. Missing standard deviations for continuous outcomes were imputed by conservatively assuming that these were equal to the largest standard deviation in the evidence base.

Due to the differences between the included trials in reporting PPG levels (with some trials using self‐monitored reporting and others using standardized meal test), PPG and PPG excursions were analysed based on SMPG curves to ensure the validity of comparisons.

As some included trials reported insulin dose in U/kg/day and others in U/day, for the purposes of data analyses, U/kg were converted to U/day by calculating the mean weight at follow‐up as baseline weight plus change in weight and multiplying this by the final weight‐adjusted insulin dose. Standard deviation was treated as missing for the trials for which this conversion was utilized.

## 3 RESULTS

### 3.1 Study selection

In total, 430 publications were identified, of which 64 publications were assessed for eligibility and 18 publications, pertaining to nine unique RCTs, were subsequently included. The updated SLR identified seven new RCTs relevant to the comparison of iGlarLixi with IDegAsp (regardless of geographical region or prior insulin exposure), in addition to the eight that were included in the previous NMA,^13^ giving a total of 15 RCTs (Table S5).

**FIGURE 1:**
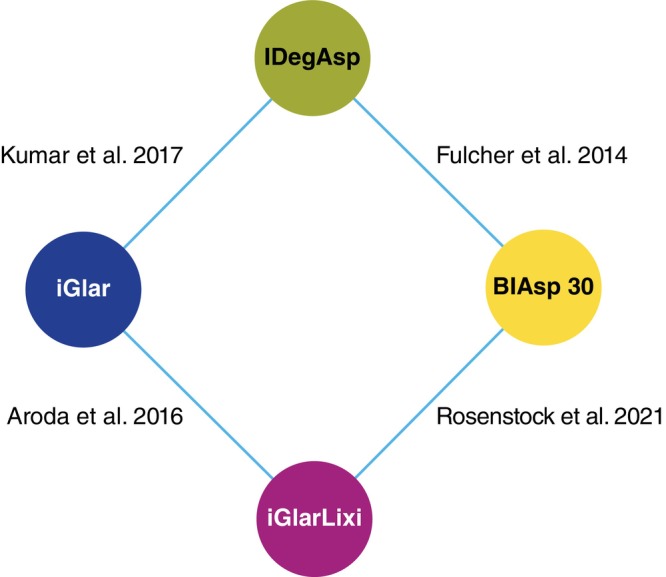
Diagram of the relationships between treatments in the included studies. Both iGlarLixi and IDegAsp are compared with BIAsp 30 and iGlar. Four non‐Asian studies evaluated treatment in the insulin‐experienced population and were thus eligible for inclusion in the updated network meta‐analysis. BIAsp 30, biphasic insulin aspart 30/70; IDegAsp, a premixed insulin of insulin degludec and insulin aspart; iGlar, insulin glargine 100 U/mL; iGlarLixi, a fixed‐ratio combination of insulin glargine 100 U/mL.

Reconciling with the evidence base from the original SLR,^13^ a total of 15 unique RCTs were identified for a global NMA. Of these 15 RCTs, studies that were conducted entirely in Asia (LixiLan JP‐O1,^19^ LixiLan JP‐O2,^20^ LixiLan JP‐L,^21^ LixiLan‐L‐CN,^22^ LixiLan‐O‐AP,^23^ BOOST INTENSIFY ALL,^24^ BOOST: JAPAN,^25^ and BOOST: INTENSIFY PREMIX/ALL 2)^26^ were excluded. Studies that included insulin‐naïve participants were also excluded from this analysis (LixiLan‐O,^8^ BOOST,^27^ and BOOST: START 1),^28^ forming a network of four RCTs (Figure 1 and Figure S1).

### 3.2 Evidence base

Two trials evaluated the use of IDegAsp (one with iGlar as comparator^10^ and one with BIAsp^11^). Two evaluated iGlarLixi (one with a comparator arm of iGlar^7^ and one with BIAsp^9^). Three of the four trials had a treatment duration of 26 weeks (Kumar et al.,^10^ Fulcher et al.^11^ and Rosenstock et al.^9^), and the remaining trial had a duration of 30 weeks.^7^ All studies included participants from intercontinental populations (Table S5).^7^, ^10^, ^11^, ^29^ All 2535 participants from the four RCTs were included in the NMA, of whom 810 received iGlarLixi and 454 received IDegAsp.

All four RCTs were open label due to the use of bespoke pre‐filled pen injectors and were of 26–30 weeks in duration. Randomization was concealed in Aroda et al.,^7^ but the methodology is not reported in the other studies. In commercial RCTs, it is common for the laboratory estimation of HbA1c to be blinded to investigators, though this was not confirmed for any of the four RCTs included.^7^ Other outcomes (e.g., SMPG, hypoglycaemia and insulin dose) were participant‐reported, and body weight was likely investigator‐measured. The primary end‐point was defined in all four RCTs. Discontinuation rates were low in the iGlarLixi studies, but there was greater leeway for bias in the IDegAsp studies. Kumar et al.^10^ reported that discontinuation was higher in the IDegAsp arm, and Fulcher et al.^11^ reported that discontinuation was higher in the comparator arm (BIAsp 30).

The baseline characteristics of the patient populations of the four trials included in the NMA are summarized in Table 1. Across the four trials, age, sex and body mass index (BMI) were generally similar, with mean age ranging from 58 to 60 years, the proportion of female participants ranging from 43%–53% and BMI from 29.3 to 31.1 kg/m^2^. There were minor differences in baseline HbA1c and fasting plasma glucose (FPG) levels, with the greatest level of variation between any two studies equivalent to one standard deviation (0.5% for HbA1c and 1.5 mmol/L for FPG).

**TABLE 1:** Patient characteristics at baseline across the trials included in the network meta‐analysis.

	Population	Treatment duration (weeks)	Randomized population (*n*)	Intervention/comparator	Concomitant therapy	Participant baseline characteristics			
						Age (mean ± SD), years	Male/female (%)	HbA1c (mean ± SD), %	BMI (mean ± SD), kg/m^2^
Kumar et al. 2017^10^ BOOST: INTENSIFY BASAL	International, insulin‐pretreated (9 countries)	26	465	IDegAsp/iGlar	Metformin ± pioglitazone ± DPP4i	57.8 ± 9.5 58.4 ± 10.1	59/41 55/45	8.3 ± 0.8 8.4 ± 1.0	30.1 ± 5.1 30.1 ± 5.3
Fulcher et al. 2014^11^ BOOST: Intensify Premix 1	International, insulin‐pretreated (10 countries)	26	447	IDegAsp/BIAsp 30	Metformin ± pioglitazone ± DPP4i	58.7 ± 9.9 58.8 ± 9.8	58/42 54/46	8.3 ± 0.8 8.4 ± 0.9	29.6 ± 4.6 29.0 ± 4.9
Aroda et al. 2016^7^ LixiLan‐L	International, insulin‐pretreated (18 countries)	30	736	iGlarLixi/iGlar	Metformin	59.6 ± 9.4 60.3 ± 8.7	45/55 49/51	8.1 ± 0.7 8.1 ± 0.7	31.3 ± 4.3 31.0 ± 4.2
Rosenstock et al. 2021^9^ SoliMix	International, insulin‐pretreated (17 countries)	26	887	iGlarLixi/BIAsp 30	Metformin ± SGLT2i	59.8 ± 10.3 59.8 ± 10.0	51/49 49/51	8.6 ± 0.7 8.6 ± 0.7	29.7 ± 4.7 30.0 ± 5.1

BIAsp 30, biphasic insulin aspart 30/70; BMI, body mass index; DPP4i, dipeptidyl peptidase‐4 inhibitor; HbA1c, glycated haemoglobin; IDegAsp, a premixed insulin of insulin degludec and insulin aspart; iGlar, insulin glargine 100 U/mL; iGlarLixi, a fixed‐ratio combination of insulin glargine 100 U/mL and lixisenatide; SD, standard deviation; SGLT2i, sodium‐glucose co‐transporter‐2 inhibitor.

In three of the four included trials,^7^, ^9^, ^10^ iGlarLixi or IDegAsp was administered once daily before a meal. In Kumar et al.,^10^ IDegAsp was most commonly injected with the main evening meal (60.2%), followed by lunchtime (18.6%) and breakfast (11.5%). In Aroda et al.,^7^ iGlarLixi was injected within 1 h before breakfast, and in Rosenstock et al.,^9^ iGlarLixi was injected within 1 h before a meal (preferably the same meal each day).

A twice‐daily administration schedule at breakfast and the main evening meal was used in one IDegAsp trial (Fulcher et al. 2014^11^; Table S6). Each trial utilized a different titration algorithm, as described in Table S6. iGlar was administered once daily in two trials,^7^, ^10^ whereas BIAsp was administered twice daily in two trials.^9^, ^11^

Postprandial glucose measurements were taken 120 min post‐meal in the two trials that evaluated iGlarLixi^7^, ^9^ and at 90 min post‐meal in the two trials with IDegAsp.^10^, ^11^ In addition, PPG was measured using a standardized meal test in Aroda et al.^7^ and was not assessed in Rosenstock et al.^9^ In the IDegAsp trials, PPG was self‐measured.

Details on prior basal insulin therapy in each study can be found in the Supplementary material.

### 3.3 Glucose control

**FIGURE 2:**
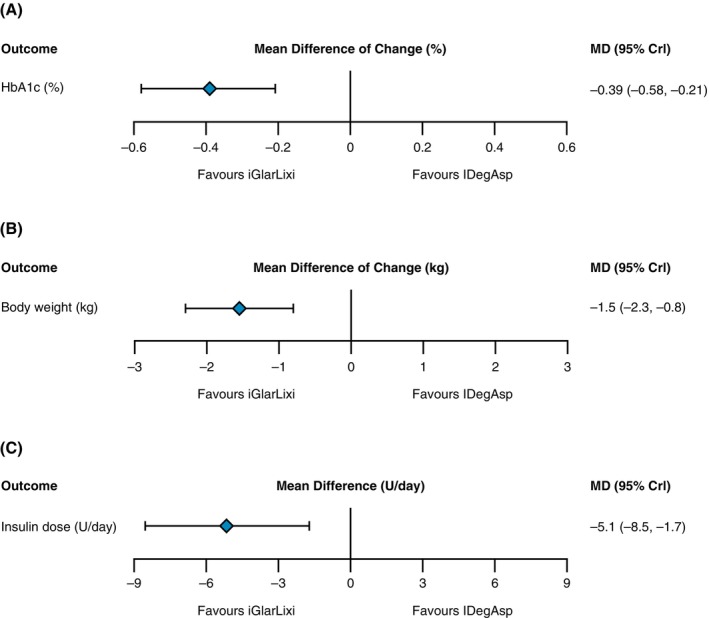
Treatment differences between iGlarLixi and IDegAsp in the insulin‐experienced population for change in (A) HbA1c, (B) body weight and (C) total insulin dose at end‐of‐study across trials included in the network meta‐analysis. CrI, credible interval; HbA1c, haemoglobin A1c; IDegAsp, a premixed insulin of insulin degludec and insulin aspart; iGlarLixi, a fixed‐ratio combination of insulin glargine 100 U/mL and lixisenatide; MD, mean difference; U, units.

The results of the NMA showed that iGlarLixi was associated with a significantly greater reduction in HbA1c compared with IDegAsp (mean difference [MD]: −0.39 [95% CrI: −0.58, −0.21] %‐units; Figure 2A). Indirect treatment comparisons showed that there was a difference in HbA1c reduction between treatments when once‐daily iGlarLixi was compared with once‐daily IDegAsp in iGlar comparison studies (MD: −0.47 [95% CrI: −0.78, −0.16] %‐units) or with twice‐daily IDegAsp in BIAsp studies (MD: −0.35 [95% CrI: −0.58, −0.21] %‐units). Treatment with iGlarLixi was also associated with a significantly greater likelihood of achieving an HbA1c of <7.0% (risk ratio: 1.42, 95% CrI: 1.18, 1.71) compared with IDegAsp. When iGlarLixi was compared with once‐daily IDegAsp via iGlar studies, the risk ratio was 1.71 (95% CrI: 1.28, 2.30), and for twice‐daily IDegAsp via BIAsp studies, the risk ratio was 1.28 (95% CrI: 0.99, 1.66).

**FIGURE 3:**
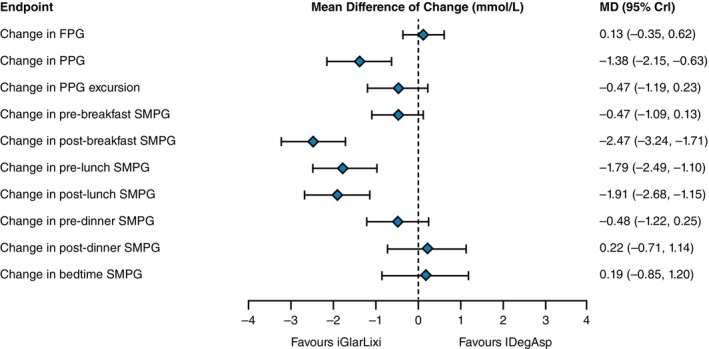
Mean differences in plasma glucose change from baseline between iGlarLixi and IDegAsp in the insulin‐experienced population from the trials included in the network meta‐analysis. CrI, credible interval; FPG, fasting plasma glucose; IDegAsp, a premixed insulin degludec and insulin aspart; iGlarLixi, a fixed‐ratio combination of insulin glargine 100 U/mL and lixisenatide; MD, mean difference; PPG, postprandial glucose; SMPG, self‐monitored plasma glucose.

No difference was found for change in FPG between iGlarLixi and IDegAsp (MD: 0.13 [95% CrI: −0.35, 0.62] mmol/L; Figure 3). iGlarLixi compared with IDegAsp was associated with greater reductions in PPG (MD: −1.38 [95% CrI: −2.15, −0.63] mmol/L), post‐breakfast SMPG (MD: −2.47 [95% CrI: −3.24, −1.71] mmol/L), pre‐lunch SMPG (MD: −1.79 [95% CrI: −2.49, −1.10] mmol/L) and post‐lunch SMPG (MD: −1.91 [95% CrI: −2.68, −1.15] mmol/L). No difference was found in post‐dinner SMPG (MD: 0.22 [95% CrI: −0.71, 1.14] mmol/L) and bedtime SMPG (MD: 0.19 [95% CrI: −0.85, 1.20] mmol/L). No difference was found for change in PPG excursion, pre‐breakfast and pre‐dinner SMPG between iGlarLixi and IDegAsp.

### 3.4 Body weight

Results of the NMA suggested a difference in body weight change between treatment groups favouring iGlarLixi versus IDegAsp (MD: −1.5 [95% CrI: −2.3, −0.8] kg; Figure 2B).

### 3.5 Insulin dose

The final insulin dose across the studies ranged between 46.7 and 97.3 U/day. Insulin doses were lower at study end in the iGlarLixi arms compared with IDegAsp arms of the included trials (MD: −5.1 [95% CrI: −8.5, −1.7] U/day; Figure 2C). The post hoc indirect treatment comparisons showed that this difference appeared to be dependent on whether iGlarLixi was compared with once‐daily IDegAsp via iGlar (MD: 0.0 [95% CrI: −4.7, 4.7] U/day) or twice‐daily IDegAsp via BIAsp (MD: −11.0 [95% CrI: −16.1, −6.0] U/day).

### 3.6 Hypoglycaemia

The definition of documented hypoglycaemia and severe hypoglycaemia differed between the four trials; details are given in Table S7. Further, the two trials that included a BIAsp control arm^9^, ^11^ reported different incidences of documented hypoglycaemia (12.9% vs. 68.9%) and severe hypoglycaemia (0.5% vs. 7.2%; Table S7) in the BIAsp arms.

Similarly, the occurrence of severe hypoglycaemia differed between the two trials using the iGlar control arms,^7^, ^10^ with rates of 1.3% and 0.3% for iGlar. Comparisons between Aroda et al.^7^ and other trials in the NMA could not be made for documented hypoglycaemia for similar reasons. These unexplained ascertainment differences were judged to render further quantitative analysis unreliable.

### 3.7 Adverse events

The risk ratio of experiencing at least one adverse event for iGlarLixi versus IDegAsp was 1.08 (95% CrI: 0.85, 1.39). The current NMA could not directly compare differences in the rates of individual categories of AEs. However, the studies included in this NMA report that the most frequent AEs for iGlarLixi were nausea (for both studies)^7^, ^9^; additionally, nervous system disorders, infections and infestations, vomiting and diarrhoea (for Aroda et al.^7^). The most commonly reported AEs for IDegAsp included nasopharyngitis, upper respiratory tract infection and headache (for both studies),^10^, ^11^ and diarrhoea and peripheral oedema in Kumar et al.^10^

There were insufficient data to make any comparisons of SAEs.

## 4 DISCUSSION

The results of this NMA found that iGlarLixi was associated with significantly greater HbA1c reduction and target achievement compared with IDegAsp in populations from non‐Asian studies who had previously received basal insulin therapy. iGlarLixi was also associated with greater reductions in PPG and a modest but favourable body weight change compared with IDegAsp. The final daily insulin dose was lower with iGlarLixi compared with twice‐daily IDegAsp in the BIAsp study analysis. Ascertainment differences across the included studies prevented the comparison of hypoglycaemia rates. While the risk of any AE did not differ, iGlarLixi had a higher rate of gastrointestinal events, as expected for a GLP‐1 RA‐containing product. Overall, this NMA suggests that there may be a clinical benefit to advancing therapy with iGlarLixi versus IDegAsp in populations from non‐Asian studies with T2D who previously received basal insulin.

The findings of the present analysis align with those previously reported in an NMA that compared iGlarLixi and IDegAsp in wider geographical and disease status populations,^13^ which suggested that iGlarLixi resulted in greater reductions in HbA1c, favourable body weight outcomes and greater target achievement versus IDegAsp. The studies included in NMAs often overlap, which is true for the previous NMA and the NMA reported here; therefore, the findings should not then be taken as statistically independent. Both found that iGlarLixi was associated with greater improvements in seven‐point SMPG profiles compared with IDegAsp, most notably at breakfast and lunchtime. This improvement in prandial glucose excursion may be attributed to the different mechanisms of action of Lixi and iAsp; the former exerts an early inhibitory effect on gastric emptying,^30^ while the latter acts directly on glucose metabolism. The dose of the meal‐time component of IDegAsp is limited by the dose of basal insulin,^31^ therefore, a higher ratio of iAsp to IDeg may lead to lower prandial excursions, perhaps at a cost of increased hypoglycaemia. However, some methodological caution is advisable when interpreting the results of this NMA due to the varied timing and measurement of postprandial SMPG, differences in the timing of iGlarLixi and IDegAsp injections and differences in the timing of IDegAsp injections between the two IDegAsp trials included in this NMA.

Severe hypoglycaemia could not be compared in the current study. The difference in incidence in the BIAsp arms between Rosenstock et al.^9^ (0.2%) and Fulcher et al.^11^ (3.1%) suggests ascertainment differences and prevents further comparison of this metric to iGlarLixi versus IDegAsp. This was also true for documented hypoglycaemia at 6.3% and 66.1% in the BIAsp arms. There is no other indication in the data that the populations studied were responding differently.

Body weight gain can be a concern for people with T2D when receiving insulin treatment.^32^ Recent treatment recommendations have emphasized that body weight control should be a priority in the management of T2D.^3^, ^33^ In the present analysis, iGlarLixi was found to be associated with a modest but favourable change in body weight at weeks 26–30 (end‐of‐study). In both iGlarLixi trials included in this analysis, iGlarLixi treatment led to a modest reduction in body weight compared with iGlar^7^ and BIAsp 30,^9^ while a body weight increase was reported in both IDegAsp trials.^10^, ^11^ Overall, the findings are consistent with reports of weight gain in comparisons of IDegAsp with basal insulin,^34^, ^35^ and body weight benefits with iGlarLixi treatment compared with basal insulin and BIAsp 30.^7^, ^9^, ^13^, ^20^, ^22^, ^23^, ^32^ There was also a lower total insulin dose at end‐of‐study for iGlarLixi when compared with twice‐daily IDegAsp.

The results of this NMA, restricted to non‐Asian studies, are consistent with the findings of a direct iGlarLixi versus IDegAsp RCT (Soli‐D), in insulin‐naïve Chinese people with T2D.^14^ The Soli‐D study demonstrated improved glycaemic control, body weight benefit, a greater proportion of participants at HbA1c target, lower total insulin dose and lower incidence and event rate of hypoglycaemia, albeit with an increased rate of gastrointestinal AEs.^14^ It is understood that the pathophysiology of T2D differs between Asian populations and non‐Asian populations,^15^, ^36^ and the ratio of iGlar and Lixi in the iGlarLixi pen‐injector used in Soli‐D (maximum 40 U/day of insulin) differs from that of the studies in the current NMA (maximum of 60 U/day of insulin).^16^, ^17^ Nevertheless, the similarity in results between the present NMA and Soli‐D may suggest that, at least qualitatively, the clinical benefits of iGlarLixi versus IDegAsp are observed in both insulin‐naïve and insulin‐experienced populations with T2D. Adverse event profiles of iGlarLixi and IDegAsp are well known and have been reported consistently in previous RCTs.^7^, ^8^, ^9^, ^10^, ^12^, ^28^ Accordingly, the lack of difference in risk ratio for AEs is unremarkable, and the rate of gastrointestinal events observed with iGlarLixi is consistent with class effects for GLP‐1 RAs.^7^, ^8^, ^19^, ^29^, ^37^ Serious adverse event rates could not be usefully compared in the current study as larger studies would be required.

A limitation of the presented NMA is that the heterogeneity of the trials included may have contributed to the differing outcomes observed in the analysis, an issue that is inherent in the methodology of NMAs.^38^ In the present study, this limitation was most notable for hypoglycaemia in the control arms of the ascertained studies. Heterogeneity between the trials may include differences in study design and patient populations. For example, the four trials utilized differing administration schedules and titration algorithms, which may have contributed to the differences in glycaemic control, body weight change, total insulin dose at end‐of‐study and hypoglycaemia. In addition, there was heterogeneity in how PPG was reported; PPG was based on SMPG profiles rather than standardized meal test data, due to the latter not being reported in Rosenstock et al., Kumar et al. and Fulcher et al.^9^, ^10^, ^11^

Only a small number of trials (four) were eligible for inclusion in the present NMA, which may not provide a comprehensive evidence base, particularly given the aforementioned heterogeneity between trials. In addition, all four of the included trials were open‐label and commercially funded, which may have led to bias in favour of the novel therapy (iGlarLixi or IDegAsp), particularly for participant‐ and investigator‐measured secondary outcomes. However, any potential bias should have been mitigated by a 26‐week duration of study and will have operated in favour of both iGlarLixi and IDegAsp versus the common comparators in this NMA (iGlar and BIAsp 30). Unfortunately, the potential bias in this NMA is unquantifiable and, therefore, uncertain when comparing studies.

The robustness of the results, especially for final insulin dose and glycaemic control, may be compromised by inconsistent reporting of prior basal insulin use across the studies. While a 26–30‐week study period may reduce baseline biases, it is unknown whether differences in basal insulin use can identify distinct populations with varying responses. Without direct head‐to‐head trials comparing iGlarLixi and IDegAsp in diverse populations, there is limited reliability of the findings. Conducting prospective randomized head‐to‐head trials with standardized outcome measures would be valuable for confirming these findings.

Strengths of this analysis include that it was based on a rigorous literature review and that the baseline characteristics between trial participants were similar. In addition, the generalizability of the results to non‐Asian populations is strengthened by the fact that the NMA comprised four global RCTs, excluding those conducted entirely in Asia.

## 5 CONCLUSIONS

Once‐daily iGlarLixi was associated with superior blood glucose control and a modest body weight benefit compared with IDegAsp in non‐Asian studies, comprising insulin‐experienced populations with T2D. The evaluation of hypoglycaemia was uncertain due to definitional and ascertainment differences of hypoglycaemia, while SAE data was not available.

## AUTHOR CONTRIBUTIONS

FL and X‐TL made substantial contributions to the study conception and design. M‐MP and X‐TL made substantial contributions to the acquisition of data. FL, KD, PS and X‐TL made substantial contributions to the analysis of data. FL, KD, PH, M‐MP and X‐TL made substantial contributions to the interpretation of data. All authors were involved in drafting the work or revising it critically for important intellectual content, and in the final approval of the version to be published. All authors have agreed to be accountable for all aspects of the work in ensuring that questions related to the accuracy or integrity of any part of the work are appropriately investigated and resolved.

## FUNDING INFORMATION

This study was funded by Sanofi.

## CONFLICT OF INTEREST STATEMENT

PH receives research support from Sanofi and consultancy fees from Eli Lilly. FL, KD and X‐TL are employees of Sanofi and may hold stocks/shares in the company. KH—advisory panel: Sanofi, Eli Lilly, Biologix, Novo Nordisk, Servier, Boehringer Ingelheim, AstraZeneca; employee: Dubai Academic Health Corporation; research support: MSD, Novo Nordisk, Sanofi, AstraZeneca; speakers bureau: Sanofi, Eli Lilly, Biologix, Novo Nordisk, MSD, Pfizer, Servier, Novartis, Boehringer Ingelheim, AstraZeneca. RM—advisory panel: Boehringer Ingelheim, Eli Lilly, Novo Nordisk; speakers bureau: Abbott, Amgen, AstraZeneca, Boehringer Ingelheim, Eli Lilly, Novo Nordisk, Sanofi, Silanes, Novartis, Bayer. KF—advisory panel: Sanofi, Novo Nordisk; speakers bureau: Sanofi, Novo Nordisk, Eli Lilly, Servier. İA has no disclosures to declare. M‐MP and PS are employees at Evidinno Outcomes Research Inc.

### PEER REVIEW

The peer review history for this article is available at https://www.webofscience.com/api/gateway/wos/peer‐review/10.1111/dom.16360.

## Supporting information


**Data S1.** Supporting information.

## Data Availability

Data are available through the corresponding author upon reasonable request. Further details on Sanofi's data‐sharing criteria, eligible studies and process for requesting access can be found at https://www.vivli.org.
