# Supplementary material for: Efficacy and safety of iGlarLixi versus IDegAsp in people with type 2 diabetes inadequately controlled with basal insulin: A systematic literature review and network meta‐analysis of non‐Asian studies
Source: Diabetes Obes Metab. 2025 Apr 2;27(6):3410–8. doi: 10.1111/dom.16360 (PMC12046479; doi:10.1111/dom.16360)
Supplement: Supplementary file 1 — Data S1. Supporting information. [file DOM-27-3410-s001.docx]

**Supplementary Material: Efficacy and safety of iGlarLixi versus IDegAsp in people with type 2 diabetes inadequately controlled with basal insulin: a systematic literature review and network meta‑analysis of non-Asian studies**

Short running title: Network meta-analysis of iGlarLixi versus IDegAsp for type 2 diabetes

Philip Home DPhil^1^; Felipe Lauand MD^2^; Khier Djaballah MD^2^; Xuan-Tony Li MSc^3^; Khadija Hafidh MD^4^; Roopa Mehta PhD^5^; Khadra Faraoun PhD^6^; İnan Anaforoğlu MD^7^; Paul Serafini BA^8^; Mir-Masoud Pourrahmat BSc^8^

^1^Translational and Clinical Research Institute, Newcastle University, Newcastle upon Tyne, UK; ^2^Sanofi, Paris, France; ^3^Sanofi, Bridgewater, New Jersey, USA; ^4^Department of Internal Medicine, Diabetology Unit, Rashid Hospital, Dubai Health Authority, Dubai, UAE; ^5^Departamento de Endocrinología y Metabolismo, UIEM, Instituto Nacional de Ciencias Médicas y Nutrición, Salvador Zubirán, México City, México; ^6^Faculty of Medicine of Oran, Université Oran 1, Algeria; ^7^Faculty of Medicine, Mehmet Ali Aydınlar Acıbadem University, Istanbul, Turkey; ^8^Evidinno Outcomes Research Inc, Vancouver, BC, Canada.

**Contents**

[**Prior basal insulin use at baseline 3**](#_Toc190166245)

[**Supplementary tables 4**](#_Toc190166246)

[**Table S1. Search strategy for Embase® via OvidSP 4**](#_Toc190166247)

[**Table S2. Search strategy for MEDLINE® via OvidSP 8**](#_Toc190166248)

[**Table S3. Search strategy for Cochrane Central Register of Controlled Trials via OvidSP 9**](#_Toc190166249)

[**Table S4. Model selection and inconsistency evaluation. 10**](#_Toc190166250)

[**Table S5. Trials identified by the systematic literature review. 13**](#_Toc190166251)

[**Table S6. Administration schedules and titration algorithms in the trials included in the network meta-analysis. 15**](#_Toc190166252)

[**Table S7. Rates and definitions of hypoglycaemia in the trials included in the network meta-analysis. 18**](#_Toc190166253)

[**Supplementary figures 21**](#_Toc190166254)

[**Figure S1. PRISMA flow diagram. 21**](#_Toc190166255)

[**References for Supplementary Material 22**](#_Toc190166256)

Information on prior basal insulin use at baseline in the four studies of the network
meta-analysis (NMA)

In SoliMix (Rosenstock et al. 2021),^1^ prior basal insulin included insulin glargine (iGlar) 100 units/mL (45.8%), iGlar 300 units/mL (21.6%), neutral protamine Hagedorn insulin (NPH; 20.7%), insulin detemir (7.3%) and insulin degludec (IDeg) (4.5%).

In LixiLan-L (Aroda et al. 2016),^2^ prior basal insulin at baseline included iGlar (64% iGlarLixi; 65% iGlar), insulin detemir (13% iGlarLixi; 15% iGlar) and NPH (23% iGlarLixi and 20% iGlar).

In BOOST: Intensify Premix 1 (Fulcher et al. 2014),^3^ prior insulin therapy included premixed or self-mixed insulin (once- or twice-daily), the type of basal insulin therapy was not reported.

In BOOST: INTENSIFY BASAL (Kumar et al. 2017),^4^ the type of basal insulin participants received prior to the study was not reported.

Supplementary tables

## **Table S1.** Search strategy for Embase® via OvidSP

| **Database: Embase <1974 to 2023 October 09>**  **Search executed: October 10, 2023** | | |
| --- | --- | --- |
| **#** | **String** | **Hits** |
| 1 | exp insulin glargine plus lixisenatide/ | 303 |
| 2 | ("ave 0010 / hoe 901" or "ave0010/hoe901" or "hoe 901 / ave 0010" or "hoe901/ave0010" or iglarlixi or "insulin glargine / lixisenatide" or "lantus/lyxumia" or lixilan or lixilan l or lixilan o or "lixisenatide / insulin glargine" or lixisenatide plus insulin glargine or "lyxumia/lantus" or soliqua or "soliqua 100/33" or suliqua).ti,ab,kw,tn. | 312 |
| 3 | exp insulin aspart plus insulin degludec/ | 189 |
| 4 | (IDegAsp or insulin aspart plus insulin degludec or "insulin degludec/insulin aspart" or "insulin degludec + insulin aspart" or "nn 5401" or "nn5401" or degludecplus or Ryzodeg).ti,ab,kw,tn. | 219 |
| 5 | or/1-4 | 595 |
| 6 | Clinical Trial/ | 1071572 |
| 7 | Randomized Controlled Trial/ | 786342 |
| 8 | controlled clinical trial/ | 471130 |
| 9 | multicenter study/ | 374482 |
| 10 | Phase 3 clinical trial/ | 69632 |
| 11 | Phase 4 clinical trial/ | 5452 |
| 12 | exp RANDOMIZATION/ | 98909 |
| 13 | Single Blind Procedure/ | 51983 |
| 14 | Double Blind Procedure/ | 211275 |
| 15 | Crossover Procedure/ | 75515 |
| 16 | PLACEBO/ | 403250 |
| 17 | randomi?ed controlled trial$.tw. | 327423 |
| 18 | rct.tw. | 54418 |
| 19 | (random$ adj2 allocat$).tw. | 55161 |
| 20 | single blind$.tw. | 31849 |
| 21 | double blind$.tw. | 245414 |
| 22 | ((treble or triple) adj blind$).tw. | 1943 |
| 23 | placebo$.tw. | 368486 |
| 24 | Prospective Study/ | 884060 |
| 25 | or/6-24 | 2954490 |
| 26 | Case Study/ | 96906 |
| 27 | case report.tw. | 544096 |
| 28 | letter/ | 1212775 |
| 29 | Editorial.pt. | 781931 |
| 30 | Letter.pt. | 1291591 |
| 31 | Note.pt. | 960147 |
| 32 | or/26-31 | 3660955 |
| 33 | 25 not 32 | 2814435 |
| 34 | 5 and 33 | 285 |
| 35 | remove duplicates from 34 | 276 |
| 36 | limit 35 to dc=20200701-20231010 | 118 |

##

## **Table S2.** Search strategy for MEDLINE® via OvidSP

| **Database: Ovid MEDLINE(R) ALL <1946 to October 09, 2023>**  **Search executed: October 10, 2023** | | |
| --- | --- | --- |
| **#** | **String** | **Hits** |
| 1 | ("ave 0010 / hoe 901" or "ave0010/hoe901" or "hoe 901 / ave 0010" or "hoe901/ave0010" or iglarlixi or "insulin glargine / lixisenatide" or "lantus/lyxumia" or lixilan or lixilan l or lixilan o or "lixisenatide / insulin glargine" or lixisenatide plus insulin glargine or "lyxumia/lantus" or soliqua or "soliqua 100/33" or suliqua).ti,ab,kw. | 143 |
| 2 | (IDegAsp or insulin aspart plus insulin degludec or "insulin degludec/insulin aspart" or "insulin degludec + insulin aspart" or "nn 5401" or "nn5401" or degludecplus or Ryzodeg).ti,ab,kw. | 106 |
| 3 | or/1-2 | 244 |
| 4 | limit 3 to dt=20200701-20231010 | 103 |
| 5 | limit 3 to rd=20200701-20231010 | 213 |
| 6 | 4 or 5 | 213 |

## **Table S3.** Search strategy for Cochrane Central Register of Controlled Trials via OvidSP

| **Database: EBM Reviews - Cochrane Central Register of Controlled Trials <September 2023>**  **Search executed: October 10, 2023** | | |
| --- | --- | --- |
| **#** | **String** | **Hits** |
| 1 | ("ave 0010 / hoe 901" or "ave0010/hoe901" or "hoe 901 / ave 0010" or "hoe901/ave0010" or iglarlixi or "insulin glargine / lixisenatide" or "lantus/lyxumia" or lixilan or lixilan l or lixilan o or "lixisenatide / insulin glargine" or lixisenatide plus insulin glargine or "lyxumia/lantus" or soliqua or "soliqua 100/33" or suliqua).ti,ab,kw. | 150 |
| 2 | (IDegAsp or insulin aspart plus insulin degludec or "insulin degludec/insulin aspart" or "insulin degludec + insulin aspart" or "nn 5401" or "nn5401" or degludecplus or Ryzodeg).ti,ab,kw. | 153 |
| 3 | or/1-2 | 301 |
| 4 | limit 3 to english | 299 |
| 5 | limit 4 to yr="2020-2023" | 99 |

##

## **Table S4.** Model selection and inconsistency evaluation.

| **Endpoint** | **DIC** | | | **Chosen Model** | **Consistency Violated** | **Tau (95% Crl)** |
| --- | --- | --- | --- | --- | --- | --- |
|  | **RE** | **FE** | **Inconsistency** |  |  |  |
| Change in HbA1c (%)* | N/A | 14.39 | 16.06 | FE | No | N/A |
| HbA1c ≤ 6.5% | N/A | N/A | N/A | N/A | N/A | N/A |
| HbA1c ≤ 7.0% | N/A | 16.63 | 15.95 | FE | No | N/A |
| Change in FPG (mmol/L)* | N/A | 14.56 | 16.06 | FE | No | N/A |
| Change in PPG (mmol/L)* | N/A | 8.02 | N/A | FE | N/A | N/A |
| Change in PPG excursion (mmol/L)* | N/A | 8.02 | N/A | FE | N/A | N/A |
| Change in pre-breakfast SMPG (mmol/L)* | N/A | 8.02 | N/A | FE | N/A | N/A |
| Change in pre-lunch SMPG (mmol/L)* | N/A | 8.02 | N/A | FE | N/A | N/A |
| Change in pre-dinner SMPG (mmol/L)* | N/A | 8.02 | N/A | FE | N/A | N/A |
| Change in post-breakfast SMPG (mmol/L)* | N/A | 8.02 | N/A | FE | N/A | N/A |
| Change in post-lunch SMPG (mmol/L)* | N/A | 8.02 | N/A | FE | N/A | N/A |
| Change in post-dinner SMPG (mmol/L)* | N/A | 8.02 | N/A | FE | N/A | N/A |
| Change in bedtime SMPG (mmol/L)* | N/A | 8.02 | N/A | FE | N/A | N/A |
| Change in body weight (kg)* | N/A | 14.29 | 16.06 | FE | No | N/A |
| Insulin dose (U)* | N/A | 24.02 | 16.07 | FE | Yes | N/A |
| Any adverse events | N/A | 7.98 | N/A | FE | N/A | N/A |
| Any serious adverse events | N/A | N/A | N/A | N/A | N/A | N/A |
| Documented hypoglycemia  (<54-56 mg/dL) | N/A | 11.98 | N/A | FE | N/A | N/A |
| Severe hypoglycemia | N/A | 32.38 | 33.91 | FE | No | N/A |
| Number of Documented hypoglycemia (< 54-56 mg/dL)† | N/A | 7.98 | N/A | FE | N/A | N/A |

*Normal model (continuous data); †Poisson model (count data); all other models were binomial (dichotomous data). Note: Consistency was violated when the DIC for the consistency model was more than 3 points lower than the DIC for the selected model. The FE model was selected when the network had only one trial per comparison (percent change in BSA at 16 weeks) or its DIC was more than 3 points lower than the RE model and the RE model was selected otherwise. AE, adverse event; CrI, Credible Interval; FE, fixed effects; FPG, fasting plasma glucose; N/A, not applicable; PPG, post-prandial glucose; RE, random effects; SAE, serious adverse event; SMPG, self-monitored plasma glucose; U, units.

## **Table S5.** Trials identified by the systematic literature review.

| **Trial name** | **Citation** | **NCT #** | **Experimental treatment** | **Phase** | **Region** | **Sample Size** | **Insulin Exposure** |
| --- | --- | --- | --- | --- | --- | --- | --- |
| SoliMix | Rosenstock et al. 2021^1^ | Not applicable | iGlarLixi | 3 | Intercontinental | 887 | Experienced |
| LixiLan-L | Aroda et al. 2016^†2^ | NCT02058160 | iGlarLixi | 3 | Intercontinental | 736 | Experienced |
| LixiLan-O-AP | Yang et al. 2022^5^ | NCT03798054 | iGlarLixi | 3 | Asia | 878 | Naïve |
| LixiLan-L-CN | Yuan et al. 2022^6^ | NCT03798080 | iGlarLixi | 3 | China | 426 | Experienced |
| LixiLan JP-L | Kaneto et al. 2020^†7^ | NCT02752412 | iGlarLixi | 3 | Japan | 512 | Experienced |
| LixiLan-O | Rosenstock et al. 2016^†8^ | NCT02058147 | iGlarLixi | 3 | Intercontinental | 1170 | Naïve |
| LixiLan JP-02 | Terauchi et al. 2020^†9^ | NCT02752828 | iGlarLixi | 3 | Japan | 521 | Naïve |
| LixiLan JP-01 | Watada et al. 2020^†10^ | NCT02749890 | iGlarLixi | 3 | Japan | 321 | Naïve |
| BOOST: INTENSIFY PREMIX/ALL 2 | Yang et al. 2019^11^ | NCT02762578 | IDegAsp | 3 | China | 543 | Experienced |
| BOOST | Kumar et al. 2016^12^ | NCT01513590 | IDegAsp | 3 | Intercontinental | 394 | Naïve |
| BOOST: Intensify Premix 1 | Fulcher et al. 2014^3^ | NCT01009580 | IDegAsp | 3 | Intercontinental | 447 | Experienced |
| BOOST INTENSIFY ALL | Kaneko et al. 2015^13^ | NCT01059812 | IDegAsp | 3 | Asia | 424 | Experienced |
| BOOST: START 1 | Kumar et al. 2016^†12^ | NCT01045707 | IDegAsp | 3 | Intercontinental | 530 | Naïve |
| BOOST: INTENSIFY BASAL | Kumar et al. 2017^†4^ | NCT01045447 | IDegAsp | 3 | Intercontinental | 465 | Experienced |
| BOOST: JAPAN | Onishi et al. 2013^†14^ | NCT01272193 | IDegAsp | 3 | Japan | 296 | Naïve |

Trials highlighted in grey were non-Asian studies that evaluated treatment in insulin-experienced populations and were included in the network meta-analysis.
^†^Denotes trials that were included in the previously published systematic literature review.^15^
IDegAsp, a premixed insulin of insulin degludec and insulin aspart; iGlarLixi, a fixed-ratio combination of insulin glargine 100 U/mL and lixisenatide; NCT, national clinical trial.

**Table S6.** Administration schedules and titration algorithms in the trials included in the network meta-analysis.

| **Administration schedule** | | | | | | | | | | | |
| --- | --- | --- | --- | --- | --- | --- | --- | --- | --- | --- | --- |
| **Trial name (citation)** | **Experimental treatment** | | **Comparator arm** | **Frequency/ timing** | | **FPG target**  **(mmol/l)** | | **Titration algorithm** | **Titration frequency** | | **Mean final insulin dose (U) (experimental arm)** |
| SoliMix  (Rosenstock et al. 2021)^1^ | iGlarLixi | | BIAsp 30 | Once-daily before a meal | | 4.4–6.1 | | C (iGlarLixi) and D (BIAsp) | Weekly | | 39.7 |
| LixiLan-L  (Aroda et al. 2016)^2^ | iGlarLixi | | iGlar | Once-daily pre-breakfast | | 4.4–5.6 | | B | Weekly | | ∼47 |
| BOOST: Intensify Premix 1 (Fulcher et al. 2014)^3^ | IDegAsp | | BIAsp 30 | Twice-daily pre-breakfast and pre-dinner | | 4.0–5.0 | | None reported | None reported | | Morning: 38  Afternoon: 52 |
| BOOST: INTENSIFY BASAL (Kumar et al. 2017)^4^ | IDegAsp | | iGlar | Once-daily pre-largest meal (usually dinner) | | 3.9–4.9 | | A | Weekly | | 60 |
| **Titration algorithm** | | | | | | | | | | | |
| **Adjustment** | | **Algorithm A** | | | **Algorithm B** | | **Algorithm C** | | | **Algorithm D** | |
| −4 U | | <3.1 mmol/L | | | – | | – | | | – | |
| −4 U to −2 U | | – | | | – | | <3.3 mmol/L | | | – | |
| −2 U | | – | | | – | | ≥3.3 to <4.4 mmol/L | | | <4.4 mmol/L | |
| −3 U | | – | | | – | | – | | | – | |
| −2 U | | 3.1–3.8 mmol/L | | | – | | – | | | – | |
| +0 U | | 3.9–4.9 mmol/L | | | – | | ≥4.4 to ≤6.1 mmol/L | | | ≥4.4 to ≤6.1 mmol/L | |
| +1 U | | – | | | – | | – | | | – | |
| +2 U | | 5.0–6.9 mmol/L | | | >5.6 and ≤7.8 mmol/L | | >6.1 to ≤7.8 mmol/L | | | 6.2–7.8 mmol/L | |
| +4 U | | 7.0–7.9 mmol/L | | | >7.8 mmol/L | | >7.8 mmol/L | | | 7.9–10.0 mmol/L | |
| +6 U | | 8.0–8.9 mmol/L | | | – | | – | | | >10 mmol/L | |
| +8 U | | ≥9.0 mmol/L | | | – | | – | | | – | |

BIAsp 30, biphasic insulin aspart 30/70; FPG, fasting plasma glucose; IDegAsp, a premixed insulin of insulin degludec and insulin aspart; iGlar, insulin glargine; iGlarLixi, a fixed-ratio combination of insulin glargine 100 U/mL and lixisenatide; U, units.

## **Table S7.** Rates and definitions of hypoglycaemia in the trials included in the network meta-analysis.

| **Trial (citation)** | **Treatment** | | **Severe hypoglycaemia  (% of participants)** | | **Symptomatic + asymptomatic hypoglycaemia (% of participants)** | |
| --- | --- | --- | --- | --- | --- | --- |
|  | **Experimental** | **Control** | **Experimental** | **Control** | **Experimental** | **Control** |
| SoliMix  (Rosenstock et al. 2021)^1^ | iGlarLixi | BIAsp | 0.20 | 0.50 | 6.30 | 12.90 |
| LixiLan  (Aroda et al. 2016)^2^ | iGlarLixi | iGlar | 1.10 | 0.30 | NA | NA |
| BOOST: Intensify Premix 1 (Fulcher et al. 2014)^3^ | IDegAsp | BIAsp | 3.10 | 7.20 | 66.10 | 68.90 |
| BOOST: INTENSIFY BASAL (Kumar et al. 2017)^4^ | IDegAsp | iGlar | 0.00 | 1.30 | 52.60 | 48.10 |
| **Trial (citation)** | **Definition of severe hypoglycaemia** | | | | | |
| SoliMix  (Rosenstock et al. 2021)^1^ | ADA level 3: a severe event characterized by altered mental and/or physical status requiring assistance for treatment of hypoglycaemia | | | | | |
| LixiLan  (Aroda et al. 2016)^2^ | Requiring another person’s assistance to actively administer carbohydrate, glucagon, or other resuscitative actions | | | | | |
| BOOST: Intensify Premix 1 (Fulcher et al. 2014)^3^ | Requiring assistance from another person | | | | | |
| BOOST: INTENSIFY BASAL (Kumar et al. 2017)^4^ | Requiring assistance from another person to treat (as defined by the ADA guidelines) with no plasma glucose level confirmation needed | | | | | |
| **Trial (citation)** | **Definition of documented hypoglycaemia** | | | | | |
| SoliMix  (Rosenstock et al. 2021)^1^ | <54 mg/dL | | | | | |
| LixiLan  (Aroda et al. 2016)^2^ | ≤70 mg/dL | | | | | |
| BOOST: Intensify Premix 1 (Fulcher et al. 2014)^3^ | <56 mg/dL | | | | | |
| BOOST: INTENSIFY BASAL (Kumar et al. 2017)^4^ | <55.9 mg/dL | | | | | |

ADA, American Diabetes Association; BIAsp, biphasic insulin aspart; iGlar, insulin glargine 100 U/mL; NA, not applicable.

Supplementary figures


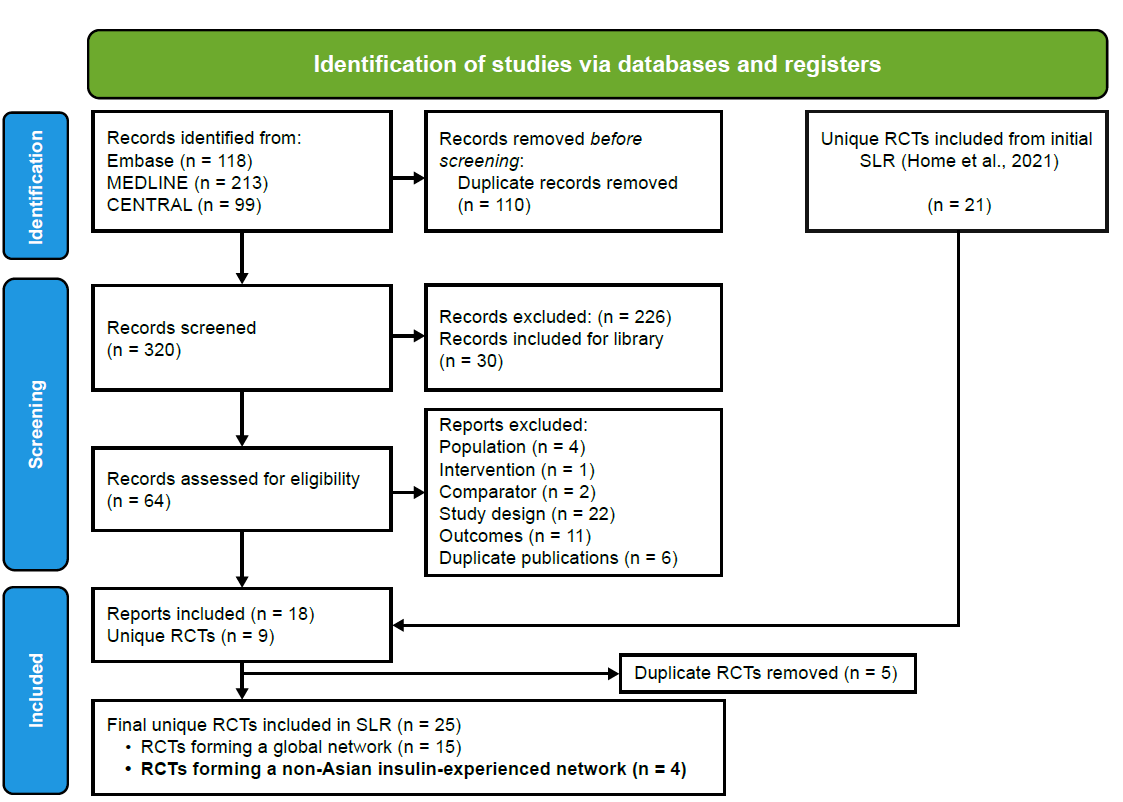


**Figure S1.** PRISMA flow diagram. PRISMA, preferred reporting items for systematic reviews and meta-analyses; RCT, randomized controlled trials; SLR, systematic literature review.^15^

References for Supplementary Material

1. Rosenstock J, Emral R, Sauque-Reyna L, et al. Advancing therapy in suboptimally controlled basal insulin-treated type 2 diabetes: clinical outcomes with iGlarLixi versus premix BIAsp 30 in the SoliMix randomized controlled trial. *Diabetes Care.* 2021;44(10):2361-2370.

2. Aroda VR, Rosenstock J, Wysham C, et al. Efficacy and safety of LixiLan, a titratable fixed-ratio combination of insulin glargine plus lixisenatide in type 2 diabetes inadequately controlled on basal insulin and metformin: the LixiLan-L randomized trial. *Diabetes Care.* 2016;39(11):1972-1980.

3. Fulcher GR, Christiansen JS, Bantwal G, et al. Comparison of insulin degludec/insulin aspart and biphasic insulin aspart 30 in uncontrolled, insulin-treated type 2 diabetes: a phase 3a, randomized, treat-to-target trial. *Diabetes Care.* 2014;37(8):2084-2090.

4. Kumar S, Jang HC, Demirağ NG, Skjøth TV, Endahl L, Bode B. Efficacy and safety of once-daily insulin degludec/insulin aspart compared with once-daily insulin glargine in participants with Type 2 diabetes: a randomized, treat-to-target study. *Diabetic medicine : a journal of the British Diabetic Association.* 2017;34(2):180-188.

5. Yang W, Dong X, Li Q, et al. Efficacy and safety benefits of iGlarLixi versus insulin glargine 100 U/mL or lixisenatide in Asian Pacific people with suboptimally controlled type 2 diabetes on oral agents: the LixiLan-O-AP randomized controlled trial. *Diabetes Obes Metab.* 2022;24(8):1522-1533.

6. Yuan X, Guo X, Zhang J, et al. Improved glycaemic control and weight benefit with iGlarLixi versus insulin glargine 100 U/mL in Chinese people with type 2 diabetes advancing their therapy from basal insulin plus oral antihyperglycaemic drugs: results from the LixiLan-L-CN randomized controlled trial. *Diabetes Obes Metab.* 2022;24(11):2182-2191.

7. Kaneto H, Takami A, Spranger R, Amano A, Watanabe D, Niemoeller E. Efficacy and safety of insulin glargine/lixisenatide fixed-ratio combination (iGlarLixi) in Japanese patients with type 2 diabetes mellitus inadequately controlled on basal insulin and oral antidiabetic drugs: The LixiLan JP-L randomized clinical trial. *Diabetes Obes Metab.* 2020;22 Suppl 4:3-13.

8. Rosenstock J, Aronson R, Grunberger G, et al. Benefits of LixiLan, a titratable fixed-ratio combination of insulin glargine plus lixisenatide, versus insulin glargine and lixisenatide monocomponents in type 2 diabetes inadequately controlled on oral agents: the LixiLan-O randomized trial. *Diabetes Care.* 2016;39(11):2026-2035.

9. Terauchi Y, Nakama T, Spranger R, Amano A, Inoue T, Niemoeller E. Efficacy and safety of insulin glargine/lixisenatide fixed-ratio combination (iGlarLixi 1:1) in Japanese patients with type 2 diabetes mellitus inadequately controlled on oral antidiabetic drugs: A randomized, 26-week, open-label, multicentre study: the LixiLan JP-O2 randomized clinical trial. *Diabetes Obes Metab.* 2020;22 Suppl 4:14-23.

10. Watada H, Takami A, Spranger R, Amano A, Hashimoto Y, Niemoeller E. Efficacy and safety of 1:1 fixed-ratio combination of insulin glargine and lixisenatide versus lixisenatide in Japanese patients with type 2 diabetes inadequately controlled on oral antidiabetic drugs: the LixiLan JP-O1 randomized clinical trial. *Diabetes Care.* 2020;43(6):1249-1257.

11. Yang W, Ma J, Hong T, et al. Efficacy and safety of insulin degludec/insulin aspart versus biphasic insulin aspart 30 in Chinese adults with type 2 diabetes: A phase III, open-label, 2:1 randomized, treat-to-target trial. *Diabetes Obes Metab.* 2019;21(7):1652-1660.

12. Kumar A, Franek E, Wise J, Niemeyer M, Mersebach H, Simó R. Efficacy and safety of once-daily insulin degludec/insulin aspart versus insulin glargine (U100) for 52 weeks in insulin-naïve patients with type 2 diabetes: a randomized controlled trial. *PLoS One.* 2016;11(10):e0163350.

13. Kaneko S, Chow F, Choi DS, et al. Insulin degludec/insulin aspart versus biphasic insulin aspart 30 in Asian patients with type 2 diabetes inadequately controlled on basal or pre-/self-mixed insulin: a 26-week, randomised, treat-to-target trial. *Diabetes Res Clin Pract.* 2015;107(1):139-147.

14. Onishi Y, Ono Y, Rabøl R, Endahl L, Nakamura S. Superior glycaemic control with once-daily insulin degludec/insulin aspart versus insulin glargine in Japanese adults with type 2 diabetes inadequately controlled with oral drugs: a randomized, controlled phase 3 trial. *Diabetes Obes Metab.* 2013;15(9):826-832.

15. Home PD, Mehta R, Hafidh KAS, et al. Efficacy and safety of iGlarLixi versus IDegAsp: Results of a systematic literature review and indirect treatment comparison. *Diabetes Obes Metab.* 2021;23(12):2660-2669.
